# Improving therapeutic synergy score predictions with adverse effects using multi-task heterogeneous network learning

**DOI:** 10.1093/bib/bbac564

**Published:** 2022-12-23

**Authors:** Yang Yue, Yongxuan Liu, Luoying Hao, Huangshu Lei, Shan He

**Affiliations:** School of Computer Science from the University of Birmingham, UK; State Key Laboratory of Agricultural Microbiology from Huazhong Agricultural University, China; School of Computer Science from the University of Birmingham, UK; YaoPharma Co., Ltd; School of Computer Science, the University of Birmingham, UK

**Keywords:** therapeutic synergy score prediction, multi-task learning, heterogeneous graph convolutional network, meta-path information aggregation for MoAs, biological networks

## Abstract

Drug combinations could trigger pharmacological therapeutic effects (TEs) and adverse effects (AEs). Many computational methods have been developed to predict TEs, e.g. the therapeutic synergy scores of anti-cancer drug combinations, or AEs from drug–drug interactions. However, most of the methods treated the AEs and TEs predictions as two separate tasks, ignoring the potential mechanistic commonalities shared between them. Based on previous clinical observations, we hypothesized that by learning the shared mechanistic commonalities between AEs and TEs, we could learn the underlying MoAs (mechanisms of actions) and ultimately improve the accuracy of TE predictions. To test our hypothesis, we formulated the TE prediction problem as a multi-task heterogeneous network learning problem that performed TE and AE learning tasks simultaneously. To solve this problem, we proposed Muthene (multi-task heterogeneous network embedding) and evaluated it on our collected drug–drug interaction dataset with both TEs and AEs indications. Our experimental results showed that, by including the AE prediction as an auxiliary task, Muthene generated more accurate TE predictions than standard single-task learning methods, which supports our hypothesis. Using a drug pair *Vincristine—Dasatinib* as a case study, we demonstrated that our method not only provides a novel way of TE predictions but also helps us gain a deeper understanding of the MoAs of drug combinations.

## Introduction

Drug combination is a more effective treatment than monotherapy for complex diseases such as cancers [1, 2]. Specifically, the use of multiple drugs that target different molecular mechanisms in the same cells not only improves the overall therapeutic effect (TE; [3, 4]), but also reduces the required concentration of each drug, which ultimately reduces the potential toxicity [5]. However, drug combinations also could cause unexpected adverse effects (AEs) such as heart failure [4, 6]. Therefore, the elucidation of both the TEs and AEs of drug combinations is critical.

In recent years, many machine learning methods, especially those based on deep learning have been developed to select drug combinations with TEs or to predict drug pairs with AEs [7, 8]. For example, the Deep Neural Network (DNN) based methods DeepDDI [9] and DeepSynergy [10] were proposed to predict polypharmacy AEs and TEs, respectively. These methods can effectively reduce corresponding wet experiment costs by predicting high-confidence drug combinations [1].

However, most of these methods only considered TE or AE information separately, which might not be optimal. Can we combine TE and AE information to obtain better prediction results for TEs and AEs? We suspect the answer is positive. The first reason is that, superficially, both tasks use the drug chemical information and drug–target interaction information as the inputs [9–11], which indicates the relatedness of the tasks. By exploiting the relatedness between the tasks, we hypothesize that we might improve their learning efficiency and prediction accuracy. More profoundly, TEs and AEs are both measurable physiological changes to drug combination treatments, hence by considering TEs and AEs together, it is more likely to reveal their underlying MoAs, e.g. their common targets and the downstream molecular networks [6], which could be beneficial to TE and AE prediction tasks.

In this paper, we limit our scope to the TE prediction and propose the following hypothesis: TE prediction could benefit from the proper use of AE information. We first formulate the TE prediction as a multi-task drug (therapeutic) synergy score regression problem [12] that uses both AE and drug–target interaction (DTI) information. Since drug–target interactions are usually modelled as a heterogeneous complex network and are also heterogeneous to AE information, we propose Muthene—a multi-task heterogeneous network learning method specifically for synergy score predictions.

The main characteristic of the proposed Muthene algorithm is its ability to capture the MoA information for both TE and AE learning. Specifically, Muthene explicitly extracts four types of meta-paths as MoAs that represent the putative interaction pathways between the involved drugs and their shared targets. To learn the extracted meta-paths, Muthene uses a heterogeneous graph convolutional network (GCN) based meta-path learning module with independent Bi-directional Gated Recurrent Unit (BiGRU) aggregators [13, 14]. For the main task, i.e. the TE prediction, Muthene first uses this meta-path learning module to extract relevant drug–target interaction information, and then trains a regressor to predict synergy scores using drug combination cell line data. Simultaneously, Muthene also conducts the AE prediction as an auxiliary task using a similar procedure as the TE prediction task, except that the learning objective is AE classification instead of synergy score regression. In essence, this auxiliary task optimizes the model parameters for better synergy score predictions through backpropagation [15].

We conducted extensive experiments on Muthene. Our experimental results showed that the synergy score prediction benefitted from the auxiliary AE prediction task, which is in line with our hypothesis. Apart from proposing and testing this novel hypothesis, our other contributions include:

- We collected and constructed a novel drug–drug interaction dataset with both TE and AE information for each sample to test our hypothesis.
- We also demonstrated that BiGRU could be a practical component for GCN to learn drug and target interaction pathway information, which might shed light on the MoAs of the drug combinations.

## Materials

### Datasets

Currently, there is no existing dataset to allow us to test the hypothesis that AE information is beneficial to synergy score predictions. To address this problem, we constructed a drug–drug interaction dataset that consisted of AEs, TEs and their relevant information based on the following datasets: DrugComb dataset (mainly for TEs; [16]), TWOSIDES dataset (mainly for AEs; [11]) and Luo *et al*. dataset (for DTIs; [17]). DrugComb is an online database that records the therapeutic effect degree of drug–drug pairs on cancer cell lines (in the form of drug–drug-cell line pairs), and the degree is measured based on four types of synergy scores separately, including Bliss [18], Highest Single Agent (HSA; [19]), Loewe [20] and Zero Interaction Potency (ZIP; [21]). The original TWOSIDES dataset has 964 kinds of AEs occurring between different drug–drug pairs. Luo *et al*. dataset is a gold standard dataset in which 1920 DTI pairs were selected.

Since DrugComb and TWOSIDES datasets contain TEs and AEs information, respectively, we needed to generate a sub-set of drug–drug pairs that had both TEs and AEs information. In other words, every existing synergy score (based on a drug–drug-cell line pair) from DrugComb should have at least one known AE. To guarantee that each (drug–drug-cell line) sample has sufficient true labels, we only selected those corresponding to the top 20 frequent AEs and the top 60 frequent cell lines from the TWOSIDES and DrugComb separately.

We then integrated DTI pairs from DrugComb, TWOSIDES and the Luo *et al*. dataset with the aforementioned sub-set, and ensured that every drug in these DTI pairs belonged to the drug set of the selected sub-set. In order to capture target–target interactions, e.g. shared pathways and cross-talk, we extracted and integrated the protein–protein interaction (PPI) network from TWOSIDES based on the targets in the selected DTI set. Next, to better capture the drug–drug interaction, we incorporated drug chemical structure information for every involved drug by generating Morgan extended-connectivity fingerprints with a radius of 3 (ECFP6), which are circular topological fingerprints commonly used in drug repurposing tasks [22, 23]. In addition, we also integrated gene expression data to depict the biological variation in the selected 60 cell lines. Specifically, for every cell line, we retrieved expression values of genes (after 0–1 normalization) that encode the drug targets from the DepMap database [24], and these targets belonged to the target set of the selected DTI set. A further illustration of the above steps is in Figure S1 of the Supplementary Material.

After the above steps with some data preprocessing, e.g. correcting naming errors and inconsistency, we obtained 11 166 drug–drug-cell line synergy scores (combinations) and 2446 drug–drug AE samples, which shared the same 106 drugs. Meanwhile, we had the corresponding 2332 DTIs, 91 785 PPIs, 106 drug ECFP6 features and 677 gene expression values for each cell line, and the overall summary of our collected dataset is shown in Table 1.

**Table 1:** The summary of our collected dataset

Data type	Total number
Synergy score types	4
Adverse effect types	20
Drug types	106
Cell line types	60
Gene types for gene expression data	677
Drug–drug-cell line synergy score sample number (4 types of synergy scores provided for each sample)	11 166
Drug–drug adverse effect sample number	2446
Drug-target interaction number	2332
Protein–protein interaction number	91 785

## Methods

### Construction of the heterogeneous therapeutic effect network

We can represent the TE-related drug–target relationships using a heterogeneous network denoted as $\mathcal{G}=(\mathcal{V},\mathcal{E},\mathcal{R})$, where ${\nu}_i\in \mathcal{V}$ denotes a node in this network, including drug (D) and protein (T); $\mathcal{E}$ is the set of edges $({\nu}_i,\mathrm{r},{\nu}_j)$ and $\mathrm{r}\in \mathcal{R}$ is the type of edges that contains drug–drug TE relationships, DTIs and PPIs. Specifically, the DTIs and PPIs are binary data, i.e. if there is an edge between the two nodes, the corresponding value is 1 (representing there is an effective edge), otherwise, it is 0. For drug–drug TE relationships, we converted the synergy score of each drug pair that was a real value to binary data using a threshold as detailed in the Supplementary Material.

To explicitly capture the MoA, e.g. the drug–target and target–target interactions (PPIs) between a drug pair for both TE and AE learning, we used the concept of meta-path schema to mimic them, which is a specific combination pattern of edges from the start (source) drug node ${\nu}_1$ to the end (central) drug node ${\nu}_{l+1}$, denoted as ${\nu_1}_{\to}^{r_1}\ {\nu_2}_{\to}^{r_2}{\dots}_{\to}^{r_l}\ {\nu}_{l+1}$ or ${\nu}_1{\nu}_2\dots{\nu}_{l+1}$. Moreover, a meta-path instance is a sequence of nodes arranged following certain kind of meta-path schemas, in which the start and end drug nodes are connected by the edges in this instance, and they are called as a pair of meta-path neighbors.

Specifically, we defined four meta-path schemas to represent (TE-related) drug–drug interaction pathways, including: $\mathrm{DTD}$, $\mathrm{DTTD}$, $\mathrm{DTTTD}$ and $\mathrm{DD}$ (TE relationships). The specific biological meanings of these schemas are shown in Table 2.

**Table 2:** Description of biological meanings of four pre-defined meta-paths

Meta-path names	Biological meanings
$\mathrm{DTD}$	The start drug shares the same protein target with the end drug
$\mathrm{DTTD}$	The start drug and end drug interact with two protein targets, which also can interact with each other
$\mathrm{DTTTD}$	The start drug and end drug interact with two protein targets, which also can interact with another protein target
$\mathrm{DD}\ (\mathrm{TE}\ \mathrm{relationships})$	The TE relationships between the start drug and end drug

Because the number of meta-path instances obtained after traversing the heterogeneous network grows exponentially with the length of the meta-path schema, to reduce the time and space complexity, we designed a heuristic sampling strategy. Specifically, we imposed an extra restriction to the 2nd protein ($\mathrm{T}$) of $\mathrm{DTTTD}$: this protein also needs to be a target of involved drugs in the network, and 50% uniform sampling ratio was used to choose the meta-path instances after applying this restriction. This could limit the meta-path instance choices for $\mathrm{DTTTD}$ into a more accurate and smaller range.

### Overview of Muthene

**Figure 1:**
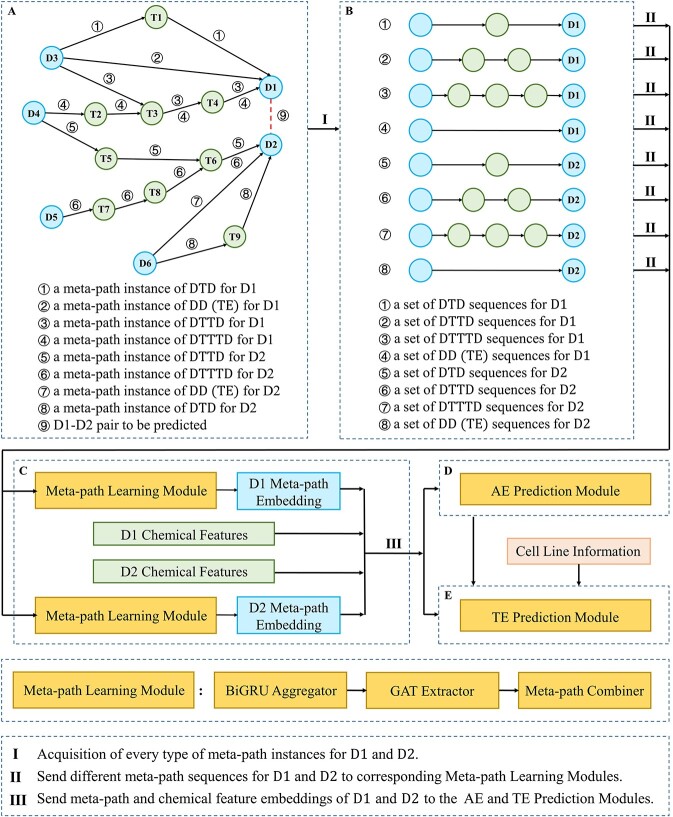
Illustration of Muthene. Take an example of predicting the synergy score between $\mathrm{D}1$ and $\mathrm{D}2$ in a certain cell line. The steps include: (**A**) Construct a heterogeneous TE network includes drug (D) and protein (T) nodes, and corresponding edges. (**B**) Acquire four types of meta-path instance sets for $\mathrm{D}1$ and $\mathrm{D}2$. (**C**) Send meta-path instance sets of $\mathrm{D}1$ and $\mathrm{D}2$ into the TE-related meta-path learning module respectively, to obtain $\mathrm{D}1$ and $\mathrm{D}2$ meta-path embeddings. Meanwhile, retrieve ECFP6 for $\mathrm{D}1$ and $\mathrm{D}2$. (**D**) Put all this information into the AE prediction module, to predict AEs between $\mathrm{D}1$ and $\mathrm{D}2$. (**E**) Put the input and output of the AE prediction module and expression data of the cell line into the TE prediction module, to calculate the final synergy score.

Muthene consists of three modules: TE-related meta-path learning, AE prediction and TE prediction modules. The illustration of Muthene is shown in Figure 1.

First, Muthene constructs a heterogeneous TE network $\mathcal{G}=(\mathcal{V},\mathcal{E},\mathcal{R})$ as detailed in section ‘Construction of the heterogeneous therapeutic effect network’. Then, for each drug–drug-cell line pair to be predicted in the network, Muthene generates all four types of meta-path instances for each drug node, to model MoAs for TE and AE learning. Second, in order to learn these meta-path instances, they are fed into the TE-related meta-path learning module to generate the meta-path embeddings of these drug nodes, which extract the interaction pathway information of the drug nodes. Third, Muthene generates the ECFP6 fingerprint for each drug node as its chemical feature embedding and then concatenates it to the drug meta-path embedding to create a drug integrated embedding. Fourth, the drug integrated embeddings and expression data of the cell line are fed to AE and TE prediction modules to predict the AE probability scores and therapeutic synergy scores of corresponding drug–drug-cell line pairs. We detailed each module below.

### TE-related meta-path learning module

#### Acquisition of meta-path instance sets

For the sake of efficiently extracting the MoA related interactive characteristics from selected meta-path instances, based on the work of [25], our TE-related meta-path learning module was designed, which can consider the information of every drug and target node in the meta-path.

To generate a meta-path node embedding (of type $t\in \mathcal{T}$) for each drug node, we needed to obtain its meta-path instance sets as the input. Take the drug node $\mathrm{D}2$ as an example, we traversed the whole heterogeneous TE network to acquire its four drug-related meta-path instance sets, i.e. $\mathrm{DTD}$, $\mathrm{DTTD}$, $\mathrm{DTTTD}$ and $\mathrm{DD}$ sets. In each set, every instance is a sequence that starts from an arbitrary (start) drug node and ends at drug node $\mathrm{D}2$ ($\mathrm{D}2$ is denoted as the end/central node of this instance), following the node-type arrangement of a specific meta-path schema. These instances in a set can construct a subgraph centered on $\mathrm{D}2$, which captures the neighborhood information of $\mathrm{D}2$ under a specific meta-path schema.

After obtaining four meta-path instance sets of a drug node, e.g. $\mathrm{D}2$, we needed to give every node in the heterogeneous TE network a feature as the initial representation in GCN, thus, after this operation, every meta-path instance was transformed into a tensor. Specifically, the representations of all drug and target nodes in the heterogeneous TE network were initialized with the node-type-specific one-hot vectors to identify every node under its node category, and these vectors were filled into every meta-path instance according to the corresponding node-type and node index. To ensure that the feature dimensions of all nodes in a meta-path instance were the same for the follow-up computation, we applied the node-type-specific transformation, for a node $i$ with node-type $t\in \mathcal{T}$, the transformation formula is as follows:

(1) \begin{equation*} {h}_i^{t\,\prime }={W}^t\bullet{h}_i^t \end{equation*}

where ${h}_i^t$ is the one-hot vector of this node, ${W}^t$ is the trainable transformation matrix for the node type $t\in \mathcal{T}$ and ${h}_i^{t\prime }$ is the aligned vector of node $i$. Because the output dimension of every transformation matrix is set to be the same, thus for $\mathrm{D}2$, after the process, $\mathrm{D}2$ will have four 3D tensors corresponding to the four original meta-path instance sets, each one contains the interactive neighborhood information of a type of meta-path schema of $\mathrm{D}2$ (for each tensor, the first dimension is the number of instances in the set, the second is the node number of the instance, the third is the output dimension); and this rule can be generalized to other drug nodes.

### Generation of drug meta-path embeddings

#### The BiGRU aggregator

 The generated meta-path instance sets of each drug node will be processed by the corresponding meta-path-specific aggregator, which generates a new representation for each meta-path instance and its end node/central node (under one type of meta-path). The generated representations from the aggregators will be used to create the meta-path-specific embeddings for the corresponding end/central node in the next step. For simplicity, a meta-path instance starting from node *i*, ending at node *j*, under the meta-path type $ m\in \mathrm{M} $ is denoted as $ {I}_{m\left(i,j\right)}$

We chose the BiGRU as the aggregator, intuitively, every ${I}_{m(i,j)}$ as a node feature sequence is reversible, for its end/central node $j$ to be predicted, ${I}_{m(i,j)}$ and ${I}_{m(j,i)}$ contain different directions of interactive pathway information for it. In other words, it is semantically meaningful to learn ${I}_{m(i,j)}$ from the start node to the end node (i.e. extraction of the forward interaction information) and learn it from the end to the start (i.e. extraction of the backward interaction information), and BiGRU could capture such bi-directional information due to its nature [13]. More specifically, we chose the final hidden state for ${I}_{m(i,j)}$ from BiGRU as the effective representation of ${I}_{m(i,j)}$, because it included all captured information from ${I}_{m(i,j)}$ on both forward and backward directions (denoted as ${h}_{m(i,j)}^f$). In addition, the updated representation of the end/central node of ${I}_{m(i,j)}$ can be obtained from the output of BiGRU (denoted as ${h}_{m(i,j)}^l$).

### The GAT extractor

To create meta-path-specific embeddings for corresponding end/central node, we adopted Graph Attention Networks (GAT), a spatial GCN [26] proposed by Velickovic *et al.* [27]. The reason to choose GAT is that it can allocate more accurate weight for every meta-path-based neighbor’s feature in the generation of the central node’s embedding.

Similar to other spatial GCNs, GAT aggregates the neighbor nodes’ features of each central node to generate the central node’s low-dimensional representation. In our GAT, the central node is chosen as the end node of every ${I}_{m(i,j)}$, the neighbor used for the central node feature aggregation is the start node (i.e. the meta-path-based neighbor of the end node) in ${I}_{m(i,j)}$ and the final hidden state for ${I}_{m(i,j)}$ (i.e. ${h}_{m(i,j)}^f$) from BiGRU is treated as the feature of the used neighbor. To illustrate this, take the generation of $\mathrm{D}2$ embedding under the meta-path $\mathrm{DTD}$ as an example: $\mathrm{D}2$ aggregates its all meta-path-based neighbors’ features in the $\mathrm{DTD}$-based subgraph, in which the feature of $\mathrm{D}2$ itself is obtained from BiGRU and the meta-path-based neighbors’ features are replaced by the corresponding meta-path instances’ representations (as these representations contain more comprehensive information than the original neighbors’ features).

More specifically, our GAT method generates embeddings of central nodes for each meta-path schema independently. For a central node $j$ under meta-path $m\in \mathrm{M}$, the formula for calculating its embedding ${h}_j^m$ is as follows:

(2) \begin{equation*} {\alpha}_{ij}^m=\frac{\exp \left(\sigma \left({a}_m^T\bullet \left[{h}_{m\left(i,j\right)}^l\Vert{h}_{m\left(i,j\right)}^f\right]\right)\right)}{\sum_{k\in{N}_J^m}\exp \left(\sigma \left({a}_m^T\bullet \left[{h}_{m\left(k,j\right)}^l\Vert{h}_{m\left(k,j\right)}^f\right]\right)\right)} \end{equation*}

where ${\alpha}_{ij}^m$ is the allocated weight for node $j$’s meta-path-based neighbor $i$ in the feature aggregation process (for generating node $j$’s embedding under meta-path $m$). $\sigma$ represents the activation function, ${a}_m^T$ is a trainable attention vector for meta-path $m$, ${N}_j^m$ represents all meta-path-based neighbors of node $j$ under meta-path $m$.

After acquiring every ${\alpha}_{ij}^m$ for node $j$, we ran a weighted aggregation to generate the embedding of node $j$ under meta-path $m$ (i.e. ${h}_j^m$). To make the GAT extractors more stable, we executed the weighted aggregation K times independently, and concatenated the output embedding from each weighted aggregation to create the new embedding, where $\Vert$ denotes the concatenation operation:

(3) \begin{equation*} {\left.{h}_j^m=\right\Vert}_{k=1}^K\sigma \left({\sum}_{i\in{N}_j^m}{\alpha}_{ij}^m\bullet{h}_{m\left(i,j\right)}^f\right) \end{equation*}

The outputs from GAT extractors are meta-path-specific embeddings. Take $\mathrm{D}2$ as an example, after running GAT, we could obtain four ($\mathrm{DTD}$, $\mathrm{DTTD}$, $\mathrm{DTTTD}$ and $\mathrm{DD}\ (\mathrm{TE}\ \mathrm{relationships})$) meta-path-specific embeddings for $\mathrm{D}2$, these embeddings will be fused to acquire the final meta-path embedding for $\mathrm{D}2$ in the next step.

### The attention mechanism for integrating meta-path-specific embeddings

After obtaining the meta-path-specific embeddings, for each drug central node, we needed to combine its embeddings based on the four meta-path types into one embedding that captured composite interaction information. To handle the heterogeneous characteristics of different meta-paths, we adopted an attention mechanism ([25, 28]; termed as the meta-path combiner) to weighed-sum the embeddings under different meta-paths automatically.

The attention mechanism starts by calculating the importance weight ${\omega}_t^m$ for fusing meta-paths of node type $t\in \mathcal{T}$ as follows:

(4) \begin{equation*} {\omega}_t^m=\frac{1}{\mid{V}_t\mid}\sum_{i\in{V}_t}{q}_t^T\bullet \tanh \left({W}_t\bullet{h}_i^m+{b}_t\right) \end{equation*}

where ${V}_t$ is the node set of type $t$, ${q}_t^T$ is the trainable attention vector for node type $t$, ${W}_t$ is the meta-path combination transformation matrix for node type $t$, ${h}_i^m$ is the embedding of node $i$ (with current node type) under meta-path $m$ defined in Equation (3) and ${b}_t$ is the trainable bias vector for node type $t$.

Next, we normalized the importance weight of every meta-path $m\in \mathrm{M}$ using softmax:

(5) \begin{equation*} {\beta}_t^m=\frac{\exp \left({\omega}_t^m\right)}{\sum_{p\in \mathrm{M}}^{\mathrm{M}}\exp \left({\omega}_t^p\right)} \end{equation*}

Utilize the normalized weight, the summarized embedding of node $i$ with type $t\in$$\mathcal{T}$ can be calculated as follows:

(6) \begin{equation*} {h}_i^t=\sum_{m\in \mathrm{M}}^{\mathrm{M}}{\beta}_t^m\bullet{h}_i^m \end{equation*}

Finally, a non-linearity is added to ${h}_i^t$, for generating the final meta-path embedding ${\mathrm{z}}_i^t$, where ${W}_P^t$ is the projection matrix of node type $t$. Based on this, the meta-path embedding of every involved drug can be obtained.

(7) \begin{equation*} {\mathrm{z}}_i^t=\sigma \left({W}_P^t\bullet{h}_i^t\right) \end{equation*}

### Integration of drug chemical information

Drug chemical features (e.g. ECFP6) are also important for drug–drug combination related predictions [29]. However, it is inappropriate to use them as the node initialization of the TE-related meta-path learning module. The reason is that, the heterogeneous TE network includes drug and target nodes, using drug chemical features in the case that the target nodes cannot be represented as such features will be harmful to the unity of the initial node embedding space [30]. Thus, we used ECFP6 of each drug node $i$ as its independent chemical feature embedding (denoted as ${z}_i^C$), which was concatenated with the corresponding meta-path embedding to produce the integrated embedding of this drug.

### The adverse effect probability score prediction module

To predict the probability of each of 20-selected AEs between a drug–drug pair, the AE prediction module combines the integrated embedding of each drug in a drug–drug pair to calculate the AE probability score:

(8) \begin{equation*} {P_{ij}}^{AE}=\sigma \left({W}_{AE}\left[{z}_i^{drug},{z}_i^C,{z}_j^{drug},{z}_j^C\right]\right) \end{equation*}

where ${W}_{AE}$ is a parameterized decoder. ${P_{ij}}^{AE}$ is a 20-dimensional vector, with each element representing the occurrence probability of a certain AE. Besides, this is a multi-label classification task, thus the binary cross entropy (BCE) loss is adopted to evaluate the difference between the predictions and corresponding ground truths, where ${x}_n$ is the predicted AE probability score for the ${n}_{th}$ sample, ${y}_n$ is the corresponding AE label ground truth and $N$ denotes the sample number.

(9) \begin{equation*} {\ell}_{BCE}= mean\left(\left\{-\left[{y}_n\cdot \mathit{\log}{x}_n+\left(1-{y}_n\right)\cdot \mathit{\log}\left(1-{x}_n\right)\right]\right\},n\in 1,\dots, N\right) \end{equation*}

### The therapeutic synergy score prediction module

In our compiled dataset, the synergy scores between drug–drug pairs are recorded based on the selected 60 cell lines, thus the gene expression data for each cell line is also added into computation to mimic the biological variation in different cell lines, for effectively distinguishing them [31].

Specifically, for obtaining cell line $k$’s gene expression input ${z}_k^{cell}$, a fully connected layer is used to reduce its original 677-dimension to a fixed dimension ${d}^{cell}$. The synergy score of the drug $i$-drug $j$-cell line $k$ pair is calculated as follows:

(10) \begin{equation*} {P_{ij k}}^{TE}={DNN}_{TE}\left[{z}_i^{drug},{z}_i^C,{z}_j^{drug},{z}_j^C,{z}_k^{cell},{P_{ij}}^{AE}\right] \end{equation*}

in which the corresponding AE prediction is explicitly added as the ${DNN}_{TE}$ input. ${DNN}_{TE}$ is the DNN having the same basic structure as the DeepSynergy by [10], for decoding the complex drug-, cell line- and AE-related input (i.e. Muthene uses the same basic structure as DeepSynergy to decode this information). Specifically, DeepSynergy was constructed based on the conic layers (where each layer had a half number of neurons compared with the last layer), and rectified linear unit (ReLU) activation [32] was additionally applied to every intermediate layer. Furthermore, to avoid the gradients were propagated from the TE prediction task back to AE prediction task in the training phase, we treated the passed ${P_{ij}}^{AE}$ as a constant.

To evaluate the model prediction error for this task, we selected the loss function mean square error (MSE) as follows, in which ${y}_n$ represents the synergy score ground truth of the ${n}_{th}$ sample, ${x}_n$ is the corresponding predicted value from the model and $N$ is the sample number.

(11) \begin{equation*} {\ell}_{MSE}= mean\left(\left\{{\left({x}_n-{y}_n\right)}^2\right\},n\in 1,\dots, N\right) \end{equation*}

To summarize, we optimized the whole framework including the three main modules in an end-to-end fashion, which made the framework better share all the effective information at once. To facilitate the training process, due to the different scale of ${\ell}_{BCE}$ and ${\ell}_{MSE}$ (i.e. each drug–drug-cell line sample will generate a BCE loss and a MSE loss for guiding the optimization, and these two losses have different value scales), we used the hyper-parameters $\alpha$ to control the weight between the two losses, for combining them as the overall optimization objective function:

(12) \begin{equation*} {\ell}_{total}=\alpha{\ell}_{MSE}+\left(1-\alpha \right){\ell}_{BCE} \end{equation*}

It is worth mentioning that, Muthene should not distinguish drug $i$-drug $j$-cell line $k$ and drug $j$-drug $i$-cell line $k$ pairs, as they have the same biological meaning. Thus, we generated each sample twice (with the different drug–drug pair order) in the training set, and the estimated values in validation and test sets were the arithmetic average of predictions of the corresponding two samples.

### Model evaluation settings

To evaluate our method, it is critical to avoid the information data leakage problem [33], which generates over-optimistic but misleading results. However, the existing model evaluation settings, i.e. randomly splitting (drug–drug-cell line) samples into training, validation and test sets suffer from this problem. To illustrate this problem, suppose we have two drug–drug-cell line pairs ${\mathrm{D}}_i-{\mathrm{D}}_j-{\mathrm{C}}_{K1}$ and ${\mathrm{D}}_i-{\mathrm{D}}_j-{\mathrm{C}}_{K2}$, if they are allocated into training and test sets separately, after training, when predicting the synergy score of ${\mathrm{D}}_i-{\mathrm{D}}_j-{\mathrm{C}}_{K2}$, the model ‘has already seen’ the information about the ${\mathrm{D}}_i-{\mathrm{D}}_j$ combination in the training phase.

To avoid this pitfall, we split drug–drug-cell line pairs based on drug–drug pairs. In other words, the drug–drug-cell line pairs with the same drug–drug pair were put into the same set (i.e. training, validation or test sets). In this case, drug–drug pairs/combinations in the test set did not occur in the training set, which avoided the information leakage problem.

Based on this setting, drug–drug-cell line pairs corresponding to 6:2:2 of all drug–drug pair varieties were allocated into training, validation and test sets, respectively, in which the training set was used for building models, the validation set was used for optimizing parameter settings and the test set was used for testing models and producing corresponding performance evaluation results. This procedure was repeated five times independently, for each time, before splitting data, our whole dataset was randomly shuffled to make different drug–drug pair varieties enter each set. We computed and reported the average evaluation metrics over the five independent repeats.

In addition, each drug–drug-cell line pair had 20 AE labels and a synergy score value. In our study, we conducted our experiments based on collected four types of synergy scores (i.e. Loewe, Bliss, HSA and ZIP), respectively. As the synergy score prediction is a regression task, we used MSE (mean square error), MAE (mean absolute error) and the Pearson correlation coefficient (abbreviated as PEARSON) as metrics to evaluate the model performance on the test set.

## Results

### AE prediction task benefits synergy score predictions

The main objective of our experiments is to test the hypothesis on whether the AE information benefits synergy score predictions in our multi-task heterogeneous network learning framework. To this end, we removed the AE prediction module from Muthene to create a variant named Muthene-AE. For further investigation, we included three representative methods DeepDDS [34], TranSynergy [1] and DeepSynergy [10]. To illustrate the difference between Muthene and these methods, we listed them in Table 3. To examine whether AE information would benefit the single-task learning methods such as DeepSynergy, we included AE binary labels of drug pairs as an extra part of its model input, and termed this variant as DeepSynergyAE. The model running environment and hyper-parameter settings are provided in Tables S1 and S2 of the Supplementary Material, and the experimental results are shown in Table 4. In addition, all involved methods were run under the same random seed.

**Table 3:** Description of the involved comparison methods

Method name	Related description
DeepDDS	DeepDDS uses GCNs and multilayer perception to encode the drug molecular graph and cell line expression data to generate drug and cell line embeddings/features respectively. Multiple fully connected layers are then used to process the concatenation of the generated drug and cell line embeddings for therapeutic effect predictions. Here following their settings, we used DeepChem [35] tool to generate the initial input graphs of GCNs for our collected drugs.
TranSynergy	TranSynergy takes drug target interaction information (as drug features) and cell line features (e.g. gene expression) as the model input, and utilizes dimension reduction layers followed by a Transformer [36] to encode them. The synergy scores are also predicted by fully connected layers.
DeepSynergy	DeepSynergy is detailed in section ‘The therapeutic synergy score prediction module’, which is a DNN-based method. In our experiments, it took the concatenation that contains ECFP6 (as drug features) of drug pairs and the cell line gene expression as the model input (the used ECFP6 is same to that used in Muthene).

**Table 4:** The averaging evaluation results of involved methods over the five independent repeats

Synergy score: Loewe	MSE	MAE	PEARSON
Muthene	**180.6258**	**8.9157**	**0.6353**
Muthene-AE	189.2802	9.0448	0.6201
DeepSynergy	203.9883	9.6107	0.5678
DeepSynergyAE	208.3976	9.6589	0.5741
DeepDDS	201.2541	9.6429	0.5714
TranSynergy	273.3114	11.5534	0.3130
Synergy score: Bliss	MSE	MAE	PEARSON
Muthene	**45.7406**	**4.3330**	**0.5503**
Muthene-AE	48.4344	4.3810	0.5155
DeepSynergy	49.6929	4.4324	0.5052
DeepSynergyAE	49.1170	4.4236	0.5093
DeepDDS	61.2581	4.7372	0.3225
TranSynergy	57.7050	4.5757	0.3595
Synergy score: HSA	MSE	MAE	PEARSON
Muthene	**30.2391**	**3.6613**	**0.4672**
Muthene-AE	31.2363	3.7435	0.4343
DeepSynergy	31.9054	3.7471	0.4111
DeepSynergyAE	31.2234	3.7430	0.4301
DeepDDS	35.7116	3.9316	0.3121
TranSynergy	35.2235	3.8679	0.2925
Synergy score: ZIP	MSE	MAE	PEARSON
Muthene	**29.2428**	**3.6736**	**0.5225**
Muthene-AE	29.6737	3.6852	0.5142
DeepSynergy	30.1284	3.7027	0.5061
DeepSynergyAE	30.5356	3.7137	0.4936
DeepDDS	34.7283	3.9232	0.4010
TranSynergy	34.1866	3.8451	0.3890

The bold data indicates the best result on current evaluation metric and synergy score type.

From the results, we found that, based on MSE, MAE and PEARSON, Muthene achieved overall better performance compared with Muthene-AE no matter on Loewe, Bliss, HSA or ZIP scores. The above clearly verified the effectiveness of our proposed multi-task framework and corresponding basic hypothesis. It is interesting to see that, the results of DeepSynergyAE were not always better than those of DeepSynergy among every type of synergy scores. This indicated that, at least the simple use of the AE information (e.g. feature concatenation) is not beneficial enough to the single-task learning method DeepSynergy.

Furthermore, we observed that among the four types of synergy scores for quantifying therapeutic effects, Loewe consistently achieved a higher Pearson regression coefficient, which indicated that it is more suitable to be a learning objective for these machine learning methods, thus, we adopted Loewe to conduct the following experiments. We also discussed the performance of the mentioned three representative methods in the Supplementary Material.

### The effectiveness of selected network components

Based on standard Muthene with the same basic hyper-parameters (shown in the Supplementary Material) and experimental settings, as well as Loewe synergy score, we did the ablation study for the important added components in this multi-task framework. First, to demonstrate the efficiency of using BiGRU as the aggregator for GCN to extract drug and target interactive pathway information from pre-defined meta-paths bi-directionally, we replaced BiGRU with Gated Recurrent Unit (GRU, i.e. unidirectional aggregator; [37]) and mean function (i.e. non-directional aggregator) to create two variants called Mut-GRU and Mut-MEAN. Specifically, for Mut-GRU, all ${h}_{m(i,j)}^l$ and ${h}_{m(i,j)}^f$ (in formulas (2) and (3)) were generated from GRU. For Mut-MEAN, ${h}_{m(i,j)}^l$ and ${h}_{m(i,j)}^f$ were obtained from the original central node’s feature described in section ‘Acquisition of meta-path instance sets’ and were obtained by averaging the features of all nodes in the corresponding meta-path instance, respectively.

We also implemented another variant, denoted as Mut-GIN, to test the effectiveness of using learnt chemical feature embeddings of drugs to replace original ECFP6 features in our method. Specifically, we used a powerful GCN named Graph Isomorphism Network (GIN) [38] to learn the molecular graph of each drug, for generating its new chemical feature embedding. The relevant detailed description can be found in the Supplementary Material, and the GIN module was trained along with other modules in an end-to-end way. The evaluation results of these variants are shown in Table 5.

**Table 5:** Ablation study results based on involved Muthene variants

Methods	MSE	MAE	PEARSON
Muthene	**180.6258**	**8.9157**	**0.6353**
Mut-GRU	191.5294	9.3234	0.6002
Mut-MEAN	186.3361	9.0604	0.6279
Mut-GIN	195.8967	9.3091	0.6019

The bold data indicates the best result on current evaluation metric.

We observed that the standard Muthene better-performed Mut-GRU and Mut-MEAN, suggesting that BiGRU did learn more useful drug and target interactive pathway information from the meta-paths. Meanwhile, the performance of Mut-GIN was inferior to that of standard Muthene, which indicated that using the drug chemical feature embedding produced from molecular graphs (by GCNs) may not be necessary in our task. In addition, we did an extra ablation study about the performance influence caused by various feature dimensions of meta-path embeddings (generated based on the heterogeneous TE network), which is shown in Figure S2 of the Supplementary Material.

### The effects of the weights between the two losses

**Figure 2:**
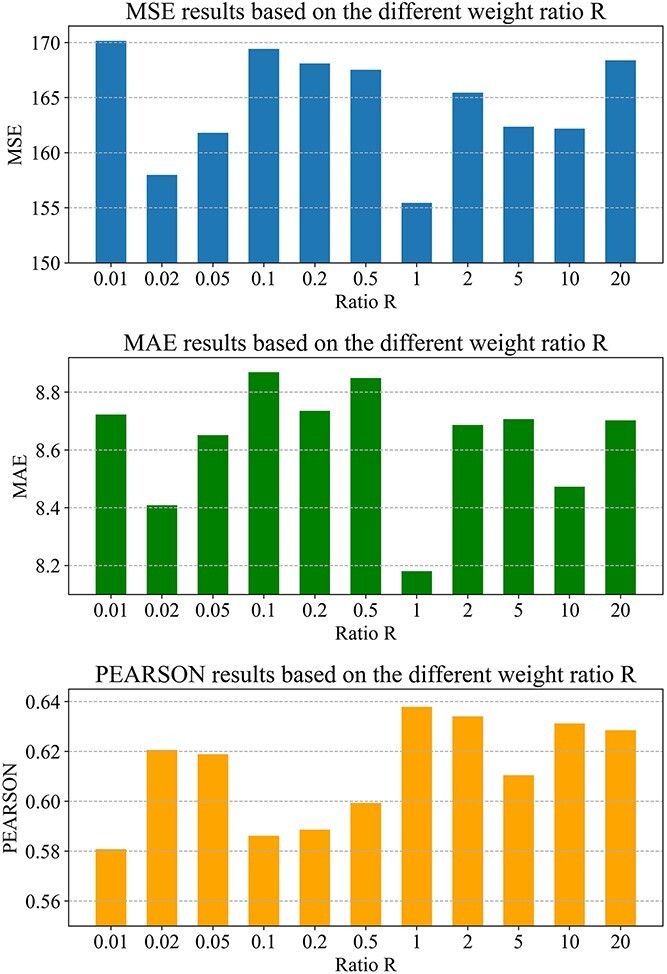
Different model evaluation results given various weight ratio R.

There is one important hyper-parameter $\alpha$ which is used to weighted combine the BCE loss and MSE loss of each sample for better optimizing Muthene. We, therefore, based on Loewe synergy score, evaluated the sensitivity of $\alpha$, through adjusting $\alpha$ while fixing the random seed and other hyper-parameters during one of the five times data splitting. The model evaluation results under different weight ratio *R* (i.e. $\frac{\alpha }{1-\alpha }$) are shown in Figure 2.

Based on the results, we can observe that, $\alpha$ was relatively sensitive, with the increase of ratio R, Muthene gradually achieved overall better Pearson regression coefficient until R reached at a threshold, and then it started to get worse. This indicated that, within a proper R scale, increasing the weight ratio R could benefit the improvement of synergy score prediction accuracy. In addition, experimentally, we found that the search range of *R* = [0.01, 0.05, 0.1, 0.5, 1, 5, 10] is suitable to find the value of *R* to demonstrate our hypothesis.

### MoAs elucidation: a case study

To demonstrate how the defined meta-paths reveal MoAs for TE predictions. We used a drug–drug pair in the test set, *Vincristine—Dasatinib* pair, which shows a significant (top ranked) drug synergy effect on Melanoma cell lines *A2058* and *UACC62*.

Since Muthene generated the meta-path embedding for each drug separately by aggregating meta-path instances of the drug, we collected the intersection of the meta-path instances between these two drugs. Not surprisingly, there is no $\mathrm{DTD}$ instance, i.e. they have different types of drug targets: *Vincristine* binds to tubulin proteins to stop the tubulins from forming microtubules, and *Dasatinib* inhibits several tyrosine kinases. However, these two drugs shared the same 114 $\mathrm{DTTD}$ instances and 340 $\mathrm{DTTTD}$ instances. From the 114 $\mathrm{DTTD}$ instances, we identified the most frequent meta-path pattern: *Vincristine* links to a tubulin protein, e.g. TUBB3, and then links to a kinase family RIPK (Receptor-interacting serine/threonine-protein kinase), e.g. RIPK1, finally links to *Dasatinib*. It is interesting to know that while both the tubulin protein and RIPK kinase families are the key regulators in cell death, their MoAs are different: Tubulin proteins cause apoptosis [39] while RIPK kinases regulate necroptosis [40]. This finding suggested that by modulating cell death through different MoAs, e.g. apoptosis and necroptosis, the *Vincristine—Dasatinib* pair provides a more effective treatment of advanced metastatic Melanoma and could overcome potential drug resistance [41].

**Figure 3:**
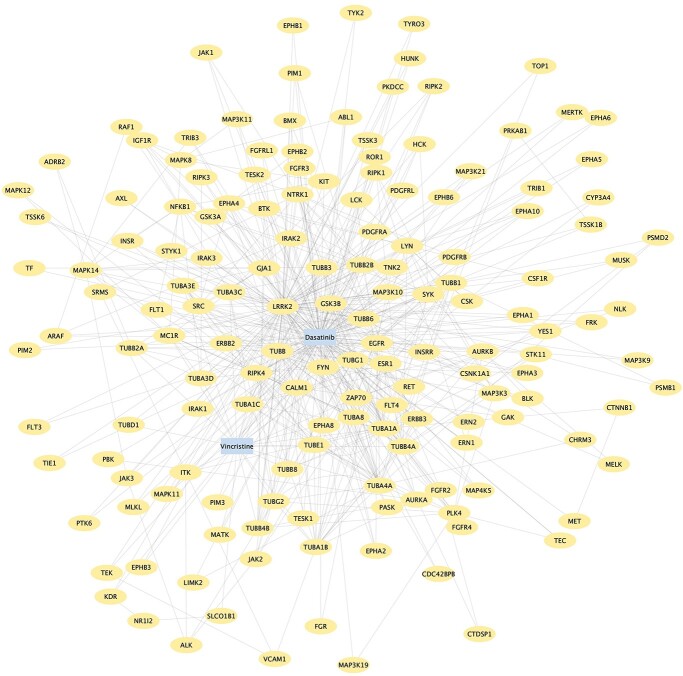
The network constituted by all $\mathrm{DTTTD}$ instances shared by *Vincristine* and *Dasatinib*.

By inspecting the 340 $\mathrm{DTTTD}$ instances, we found another interesting MoA hypothesis. Among all the targets of the $\mathrm{DTTTD}$ instances, Leucine-rich repeat kinase 2 (LRRK2), a target that bridges tubulin targets of *Vincristine* and kinase targets of *Dasatinib* (e.g. RIPK1 and RIPK4), appears more frequently than other (target) nodes. A Betweenness Centrality analysis of the network generated from these $\mathrm{DTTTD}$ instances (Figure 3) confirmed that LRRK2 has the highest Betweenness Centrality, which means that it is the central or hub node of the network. From a complex network perspective, a central node should play an important role in network functions, i.e. Melanoma progression in our case. However, LRRK2 is a well-known gene associated with Parkinson’s disease [42]. How does LRRK2 appear in the $\mathrm{DTTTD}$ instances as the putative MoAs for treating Melanoma? A meta-analysis showed that Parkinson’s disease patients have a high risk of getting Melanoma [43], and a subsequent genomic study confirmed the mutation of LRRK2 is associated with Melanoma [44]. Interestingly, our Muthene algorithm independently uncovered this association (through using the meta-path schema). More importantly, Muthene also hypothesized that the high synergy between *Vincristine—Dasatinib* pair could be explained by the indirect modulation of LRRK2 by *Vincristine*, which subsequently enhances the regulation of the kinase targets of *Dasatinib*.

To summarize, this case study shows that our Muthene algorithm can not only predict the drug synergy effect but also shed light on the potential MoAs using meta-paths.

## Conclusion

Based on the hypothesis that the therapeutic effect prediction task could benefit from adverse effect information, we formulated the therapeutic synergy score prediction as a multi-task heterogeneous network learning problem that simultaneously learned synergy score and adverse effect predictions for drug–drug pairs. To solve this problem, we proposed Muthene, a multi-task heterogeneous network learning method specifically for synergy score predictions.

To capture MoA information for the both predictions, which is critical for rational drug repurposing, Muthene extracted meta-paths to represent the underlying chemical and/or molecular network interactions. Our experimental results showed that Muthene generated more accurate synergy score predictions than standard single-task learning methods, which supports our hypothesis. Our method is a novel way to predict TEs based on multiple sources of information, including utilizing AE information, which can effectively improve the TE prediction accuracy. More significantly, using a drug pair *Vincristine—Dasatinib* as a case study, Muthene generated a few interesting hypotheses about the underlying MoAs of drug combinations. For example, Muthene revealed the synergistic effects of apoptosis and necroptosis caused by *Vincristine* and *Dasatinib*, respectively, and the additive effect exerted by LRRK2, an unexpected cancer target indirectly modulated by *Vincristine*.

However, we must admit that the meta-paths are an over-simplification of the complex molecular and chemical networks, which might not reflect the complexity of interactions between drugs and targets. We plan to introduce more semantically meaningful meta-paths from external data sources to further improve the performance of Muthene in terms of the TE prediction and MoA illustration.

**Key Points**

- We proposed a hypothesis based on previous clinical observations that by learning the shared mechanistic commonalities between adverse effects (AEs) and therapeutic effects (TEs), we could learn the underlying MoAs (mechanisms of actions) and ultimately improve the accuracy of TE predictions.
- To test our hypothesis, we formulated the TE prediction problem as a multi-task heterogeneous network learning problem that performed TE and AE learning tasks simultaneously, and solved this problem by a novel heterogeneous graph convolutional network based method Muthene.
- To better capture the MoAs of drug combinations for both TE and AE learning, we explicitly pre-defined four meta-paths with different biological meanings. To evaluate our method, we collected and constructed a novel drug–drug interaction dataset that had both TE and AE information for each sample.
- The experimental results demonstrated the feasibility and effectiveness of our hypothesis.

## Supplementary Material

Supplementary_Material_bbac564

## Data Availability

The source data and code of Muthene are available via: https://github.com/arantir123/HNEMA.

## Author Biographies

**Yang Yue** is a PhD student of the School of Computer Science from the University of Birmingham, UK. His research interests include bioinformatics, machine learning and data mining.

**Yongxuan Liu** is a PhD student of the State Key Laboratory of Agricultural Microbiology from Huazhong Agricultural University, China. His research interests include synthetic biology and pathogenic microbiology of Pseudomonas.

**Luoying Hao** is a PhD student of the School of Computer Science from the University of Birmingham, UK. Her research interests include medical image analysis, video understanding and deep learning.

**Huangshu Lei** is the chief scientist of YaoPharma Co., Ltd. His research interests are drug discovery and organic chemistry.

**Shan He** is an associate professor at the School of Computer Science, the University of Birmingham. His research interests are machine learning, evolutionary algorithms, network medicine and drug discovery.

## Notes

The data and source code are available at: https://github.com/arantir123/HNEMA.

## References

[1] Liu Q , Xie L. TranSynergy: mechanism-driven interpretable deep neural network for the synergistic prediction and pathway deconvolution of drug combinations. PLoS Comput Biol 2021;17(2):e1008653. 33577560 10.1371/journal.pcbi.1008653 PMC7906476

[2] Jia J , Zhu F, Ma X, et al. Mechanisms of drug combinations: interaction and network perspectives. Nat Rev Drug Discov 2009;8(2):111–28. 19180105 10.1038/nrd2683

[3] Csermely P , Korcsmáros T, Kiss HJM, et al. Structure and dynamics of molecular networks: a novel paradigm of drug discovery: a comprehensive review. Pharmacol Ther 2013;138(3):333–408. 23384594 10.1016/j.pharmthera.2013.01.016 PMC3647006

[4] Jiang P , Huang S, Fu Z, et al. Deep graph embedding for prioritizing synergistic anticancer drug combinations. Comput Struct Biotechnol J 2020;18:427–38. 32153729 10.1016/j.csbj.2020.02.006 PMC7052513

[5] Sun W , Sanderson PE, Zheng W. Drug combination therapy increases successful drug repositioning. Drug Discov Today 2016;21(7):1189–95. 27240777 10.1016/j.drudis.2016.05.015 PMC4907866

[6] Zhang P , Wang F, Hu J, et al. Exploring the relationship between drug side-effects and therapeutic indications. Am Med Inform Assoc AMIA Annual Symposium Proceedings 2013;2013:1568. PMC3900166 24551427

[7] Chen X , Yan CC, Zhang X, et al. Drug–target interaction prediction: databases, web servers and computational models. Brief Bioinform 2016;17(4):696–712. 26283676 10.1093/bib/bbv066

[8] Wang H , Pujos-Guillot E, Comte B, et al. Deep learning in systems medicine. Brief Bioinform 2021;22(2):1543–59. 33197934 10.1093/bib/bbaa237 PMC8382976

[9] Ryu JY , Kim HU, Lee SY. Deep learning improves prediction of drug–drug and drug–food interactions. Proc Natl Acad Sci 2018;115(18):E4304–11. 29666228 10.1073/pnas.1803294115 PMC5939113

[10] Preuer K , Lewis RPI, Hochreiter S, et al. DeepSynergy: predicting anti-cancer drug synergy with Deep Learning. Bioinformatics 2018;34(9):1538–46. 29253077 10.1093/bioinformatics/btx806 PMC5925774

[11] Zitnik M , Agrawal M, Leskovec J. Modeling polypharmacy side effects with graph convolutional networks. Bioinformatics 2018;34(13):i457–66. 29949996 10.1093/bioinformatics/bty294 PMC6022705

[12] Zhang D , Shen D. Alzheimer's disease neuroimaging initiative. Multi-modal multi-task learning for joint prediction of multiple regression and classification variables in Alzheimer's disease. Neuroimage 2012;59(2):895–907. 21992749 10.1016/j.neuroimage.2011.09.069 PMC3230721

[13] Jiao Z , Sun S, Sun K. Chinese lexical analysis with deep bi-gru-crf network. arXiv preprint arXiv:180701882 2018. https://arxiv.org/abs/1807.01882.

[14] Purkayastha S , Mondal I, Sarkar S, et al. Drug-drug interactions prediction based on drug embedding and graph auto-encoder/. 2019 IEEE 19th International Conference on Bioinformatics and Bioengineering (BIBE). IEEE, 2019: 547–52.

[15] Peng W , Tang Q, Dai W, et al. Improving cancer driver gene identification using multi-task learning on graph convolutional network. Brief Bioinform 2022;23(1):bbab432. 34643232 10.1093/bib/bbab432

[16] Zheng S , Aldahdooh J, Shadbahr T, et al. DrugComb update: a more comprehensive drug sensitivity data repository and analysis portal. Nucleic Acids Res 2021;49(W1):W174–84. 34060634 10.1093/nar/gkab438 PMC8218202

[17] Luo Y , Zhao X, Zhou J, et al. A network integration approach for drug-target interaction prediction and computational drug repositioning from heterogeneous information. Nat Commun 2017;8(1):1–13. 28924171 10.1038/s41467-017-00680-8 PMC5603535

[18] Bliss CI . The toxicity of poisons applied jointly 1. Ann Appl Biol 1939;26(3):585–615.

[19] Tan X , Hu L, Luquette LJ, et al. Systematic identification of synergistic drug pairs targeting HIV. Nat Biotechnol 2012;30(11):1125–30. 23064238 10.1038/nbt.2391 PMC3494743

[20] Loewe S . The problem of synergism and antagonism of combined drugs. Arzneimittelforschung 1953;3(6):285–90. 13081480

[21] Yadav B , Wennerberg K, Aittokallio T, et al. Searching for drug synergy in complex dose–response landscapes using an interaction potency model. Comput Struct Biotechnol J 2015;13:504–13. 26949479 10.1016/j.csbj.2015.09.001 PMC4759128

[22] Rogers D , Hahn M. Extended-connectivity fingerprints. J Chem Inf Model 2010;50(5):742–54. 20426451 10.1021/ci100050t

[23] Landrum G . RDKit: a software suite for cheminformatics, computational chemistry, and predictive modeling. Greg Landrum 2013.

[24] Tsherniak A , Vazquez F, Montgomery PG, et al. Defining a cancer dependency map. Cell 2017;170(3):564, e16–76. 28753430 10.1016/j.cell.2017.06.010 PMC5667678

[25] Fu X , Zhang J, Meng Z, et al. Magnn: Metapath aggregated graph neural network for heterogeneous graph embedding. Proceedings of The Web Conference 2020. 2020: 2331–41.

[26] Kipf TN, Welling M. Semi-Supervised Classification with Graph Convolutional Networks. In: 5th International Conference on Learning Representations, ICLR 2017, Toulon, France, April 24–26, 2017, Conference Track Proceedings. OpenReview.net, 2017.

[27] Velickovic P , Cucurull G, Casanova A, et al. Graph attention networks. Stat 2017;1050:20.

[28] Wang X, Ji H, Shi C, et al. Heterogeneous Graph Attention Network. In: The World Wide Web Conference, WWW '19. Association for Computing Machinery, New York, USA, 2019, 2022 32.

[29] Huang K, Xiao C, Hoang T, et al. Caster: predicting drug interactions with chemical substructure representation. In: Proceedings of the Thirty-Fourth AAAI Conference on Artificial Intelligence. AAAI Press, Washington, DC, USA, 2020;34(01):702 9.

[30] Yu Y , Huang K, Zhang C, et al. SumGNN: multi-typed drug interaction prediction via efficient knowledge graph summarization. Bioinformatics 2021;37(18):2988–95. 10.1093/bioinformatics/btab207 PMC10060701 33769494

[31] Talloen W , Clevert DA, Hochreiter S, et al. I/NI-calls for the exclusion of non-informative genes: a highly effective filtering tool for microarray data. Bioinformatics 2007;23(21):2897–902. 17921172 10.1093/bioinformatics/btm478

[32] Nair V, Hinton GE. Rectified linear units improve restricted boltzmann machines. In: Proceedings of the 27th International Conference on International Conference on Machine Learning, ICML'10. Omnipress, Madison, USA, 2020, 807–14.

[33] Kaufman S , Rosset S, Perlich C, et al. Leakage in data mining: formulation, detection, and avoidance. ACM Trans Knowl Disc Data (TKDD) 2012;6(4):1–21.

[34] Wang J , Liu X, Shen S, et al. DeepDDS: deep graph neural network with attention mechanism to predict synergistic drug combinations. Brief Bioinform 2022;23(1):bbab390. 34571537 10.1093/bib/bbab390

[35] Ramsundar B , Eastman P, Walters P, et al. Deep Learning for the Life Sciences: Applying Deep Learning to Genomics, Microscopy, Drug Discovery, and More. O'Reilly Media, 2019.

[36] Vaswani A , Shazeer N, Parmar N, et al. Attention is all you need. Adv Neural Inf Proc Syst 2017;30.

[37] Cho K, van Merrienboer B, Gülçehre Ç, et al. Learning Phrase Representations using RNN Encoder-Decoder for Statistical Machine Translation. In: The 2014 Conference on Empirical Methods on Natural Language Processing, EMNLP 2014, Doha, Qatar, October 25–29, 2014, Conference Track Proceedings. OpenReview.net, 2014.

[38] Xu K, Hu W, Leskovec J, et al. How Powerful are Graph Neural Networks? In: 7th International Conference on Learning Representations, ICLR 2019, New Orleans, USA, May 6–9, 2019, Conference Track Proceedings. OpenReview.net, 2019.

[39] Gan PP , McCarroll JA, Po'uha ST, et al. Microtubule dynamics, mitotic arrest, and apoptosis: drug-induced differential effects of βIII-TubulinβIII-Tubulin, microtubule dynamics, and mitotic arrest. Mol Cancer Ther 2010;9(5):1339–48. 20442307 10.1158/1535-7163.MCT-09-0679

[40] Heckmann BL , Tummers B, Green DR. Crashing the computer: apoptosis vs. necroptosis in neuroinflammation. Cell Death Diff 2019;26(1):41–52. 10.1038/s41418-018-0195-3 PMC6294765 30341422

[41] Su Z , Yang Z, Xu Y, et al. Apoptosis, autophagy, necroptosis, and cancer metastasis. Mol Cancer 2015;14(1):1–14. 25743109 10.1186/s12943-015-0321-5 PMC4343053

[42] Zimprich A , Biskup S, Leitner P, et al. Mutations in LRRK2 cause autosomal-dominant parkinsonism with pleomorphic pathology. Neuron 2004;44(4):601–7. 15541309 10.1016/j.neuron.2004.11.005

[43] Liu R , Gao X, Lu Y, et al. Meta-analysis of the relationship between Parkinson disease and melanoma. Neurology 2011;76(23):2002–9. 21646627 10.1212/WNL.0b013e31821e554e PMC3116643

[44] Agalliu I , San Luciano M, Mirelman A, et al. Higher frequency of certain cancers in LRRK2 G2019S mutation carriers with Parkinson disease: a pooled analysis. JAMA Neurol 2015;72(1):58–65. 25401981 10.1001/jamaneurol.2014.1973 PMC4366130

