# Supplementary material for: Improving therapeutic synergy score predictions with adverse effects using multi-task heterogeneous network learning
Source: Brief Bioinform. 2022 Dec 23;24(1):bbac564. doi: 10.1093/bib/bbac564 (PMC9851313; doi:10.1093/bib/bbac564)
Supplement: Supplementary_Material_bbac564 [file supplementary_material_bbac564.docx]

Supplementary Information (SI)

**Improving Therapeutic Synergy Score Predictions with Adverse Effects using Multi-task Heterogeneous Network Learning**

Yang Yue^a^, Yongxuan Liu^b^, Luoying Hao^a^, Huangshu Lei^c^, and Shan He^a,^^[[1]](#footnote-1)^*

^a^ Centre for Computational Biology, School of Computer Science, The University of Birmingham, Edgbaston, Birmingham, B15 2TT, UK

^b^ State Key Laboratory of Agricultural Microbiology, Huazhong Agricultural University, Wuhan, 430070, China

^c^ YaoPharma Co., Ltd., 100 Xingguang Avenue, Renhe Town, Yubei District, Chongqing, 401121, China

In the Supplementary Material, we mainly introduce six sections:

In the section 1, we explain the steps for retrieving our collected dataset with more details (corresponding to the **Datasets** section of the manuscript).

In the section 2, we introduce the selection of the threshold for drug-drug TE relationships (corresponding to the **Construction of the heterogeneous therapeutic effect network** section of the manuscript).

In the section 3, we introduce the model running environment and hyper-parameter settings of involved methods (corresponding to the **AE prediction task benefits synergy score predictions** section of the manuscript).

In the section 4, we discuss the performance of the involved three representative comparison methods (corresponding to the **AE prediction task benefits synergy score predictions** section of the manuscript).

In the section 5, we introduce how to implement the variant Mut-GIN of Muthene (corresponding to **The effectiveness of selected network components** section of the manuscript).

In the section 6, we introduce the ablation study about the dimension for meta-path embeddings (corresponding to **The** **effectiveness of selected network components** section of the manuscript).

1. **The illustration of steps of collecting our dataset**


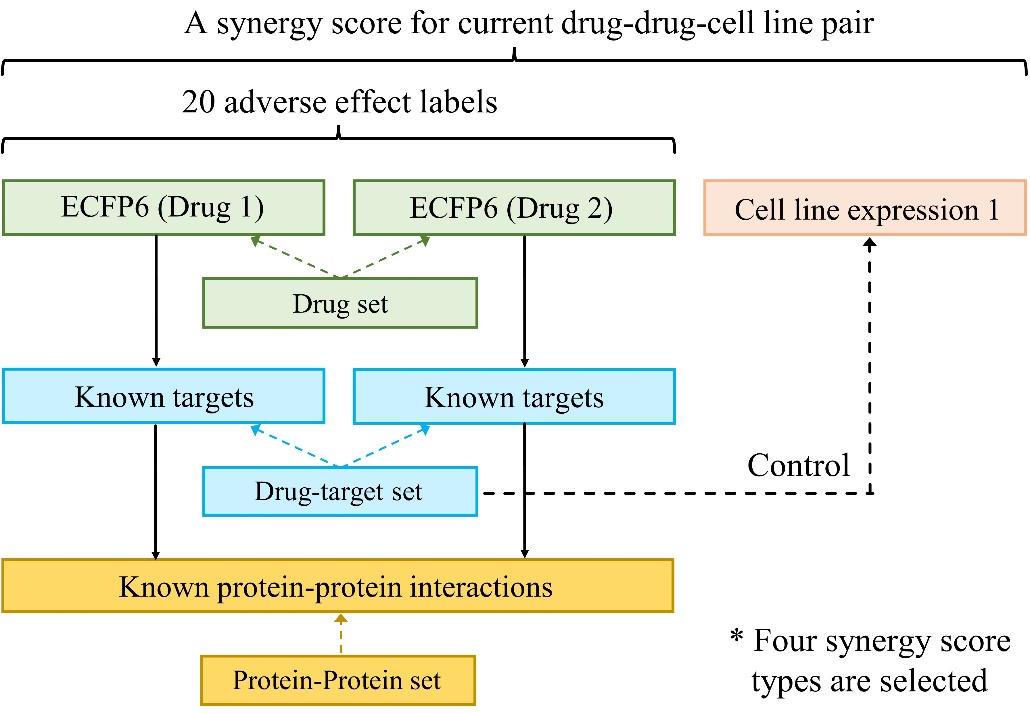


**Fig. S1.** The illstruation of collecting relevant data for a drug-drug-cell line pair.

As illustrated in Figure S1, to retrieve relevant data for a drug-drug-cell line pair (combination), for example, $D1-D2-C1$, we first make this sample equipped with a Loewe synergy score and 20 adverse effect labels (with at least one known/positive value). Next, retrieve ECFP6 corresponding to $D1$ and $D2$ to characterize their chemical structural property. Next, obtain targets of $D1$ and $D2$ from the drug-target set (based on the DrugComb dataset, TWOSIDES dataset, and Luo et al. dataset). Then, from the PPI set (based on the TWOSIDES dataset), obtain protein-protein interaction (PPI) information of the targets retrieved in the last step. Finally, from the DepMap database, retrieve expression data of genes that belong to the targets in the drug-target set. All this information will be utilized by Muthene for predicting the synergy score of $D1-D2-C1$.

1. **Threshold selection for drug-drug TE relationships**

For drug-drug TE relationships, we give an effective edge between drug $i$ and drug $j$ based on a threshold originated from corresponding therapeutic synergy scores (Peng, et al., 2021), the detailed steps are as follows.

we first calculate the value at the 95% percentile of the standard normal distribution, which is approximately 1.64. Then, we calculate the overall mean value ($\mu$) and variance ($var$) of synergy score samples. Next, for each synergy score $s_{ijk}$ that represents the therapeutically synergistic effect degree between drug node$i$, drug node $j$, and cell line $k$ pair, we calculate its $z-score=(s_{ijk}-\mu)/\sqrt{var}$. If any of $s_{ijk}$ between drug node$i$ and drug node $j$ is larger than or equal to 1.64, the drug $i$-drug $j$ TE relationship value is set to 1, indicating that there exists an effective edge, otherwise it is 0.

1. **Model running environment and hyper-parameter settings**

To demonstrate our hypothesis, we use Python 3.6.13 and Pytorch 1.10.2 to build the framework of involved models, all models are run on the same server with the configuration of Linux 4.18, CUDA tool kit 11.3.1, Intel Xeon Platinum 8360Y, and NVIDIA A100-SXM. The basic hyper-parameters of Muthene, DeepSynergy, and their variants are shown in Tables S1 and S2. For DeepDDS and TranSynergy, we adopt their recommended basic hyper-parameters in their original papers.

**Table S1.** The hyper-parameters shared by all Muthene variants in the comparison experiment.

| Hyper-parameter names | Hyper-parameter values |
| --- | --- |
| Meta-path embedding dimension | 64 |
| Weighted aggregation times K in GAT | 8 |
| Cell line feature dimension $d^{cell}$ | 64 |
| Neuron numbers of conic layers in ${DNN}_{TE}$ | 2048, 1024, 512 |

**Table S2.** The hyper-parameters shared by all DeepSynergy variants in the comparison experiment.

| Hyper-parameter names | Hyper-parameter values |
| --- | --- |
| Neuron numbers of conic layers | 2048, 1024, 512 |

1. **Discussion of involved three representative methods**

For the mentioned three representative methods in the comparison, DeepSynergy achieved overall higher prediction accuracy compared with DeepDDS and TranSynergy. We thought the main reason was that TranSynergy requires the drug target interaction information as drug features, in which involved targets/genes should be consistent with the genes included in cell line features. The performance of TranSynergy would be limited when the lack of interactive information of above specific target/genes for involved drugs (Liu and Xie, 2021). While in our dataset, 11 out of 106 drugs do not include the interactions with these targets/genes, leading to the sub-optimal results of the method. For DeepDDS, its drug features are generated by GCNs (from learning drug molecular graphs). However, we used a stricter model evaluation setting (i.e., drug pairs in the test/validation set will not occur in the training set), in this case, GCNs cannot produce effective drug features for the drugs that have not seen in the training set (e.g., in one fold of the independent repeats, 14/16 types of drugs were in the validation/test set but not in the training set), making it difficult to be generalized to drug-drug-cell line pairs corresponding to these drugs.

1. **Detailed description of Mut-GIN**

Drug chemical feature information is important for drug-drug combination related predictions (Huang, et al., 2020). To test the effectiveness of using the learnt drug chemical feature embeddings to replace commonly used ECFP6 features, we design a GCN-based module to produce the new embeddings. Specifically, for each drug node, we acquire its molecular graph with atoms as nodes, chemical bonds as edges, and numerical encoding of the atomic number as the initial node embedding. The Graph Isomorphism Network (GIN) is adopted to learn each molecular graph, as it has close distinguishing capacity to the Weisfeiler-Lehman (WL) test in learning graph structures and has already been demonstrated to have better performance in many bioinformatics graph classification tasks compared with the vanilla GCN (Xu, et al., 2018).

Formally, for a molecular graph of a drug, the embedding of a atom node $i$ at layer $l+1$ is updated as follows:

|  | $h_{i}^{(l+1)}={\sigma(MLP}^{l+1}(\left( 1+\epsilon\right)h_{i}^{\left( l \right)}+SUM(\{h_{j}^{l},j\in N(i)\})))$ | (1) |
| --- | --- | --- |

where $\epsilon$ is a trainable parameter and $N(i)$ is the neighbor set of atom node $i$. After stacking multiple GIN layers, all atom embeddings of a drug are aggregated through the global mean pooling (i.e., $READOUT$) function, to generate the chemical feature embedding for the compound $c$:

|  | $z_{c}^{G}=READOUT(\{h_{i}^{\left( l+1 \right)}\vert i\in G\})$ | (2) |
| --- | --- | --- |

This module will be trained along with the main modules of Muthene in an end-to-end way for joint optimization (denoted as Mut-GIN).

1. **Ablation study for dimension of meta-path embeddings**

In order to investigate the performance influence caused by different choices of meta-path embedding dimensions, based on the Loewe synergy score, we report the performance change of Muthene when changing the meta-path embedding dimension while fixing other hyper-parameters (during one of the five times data splitting), and the results are shown in Figure S2.


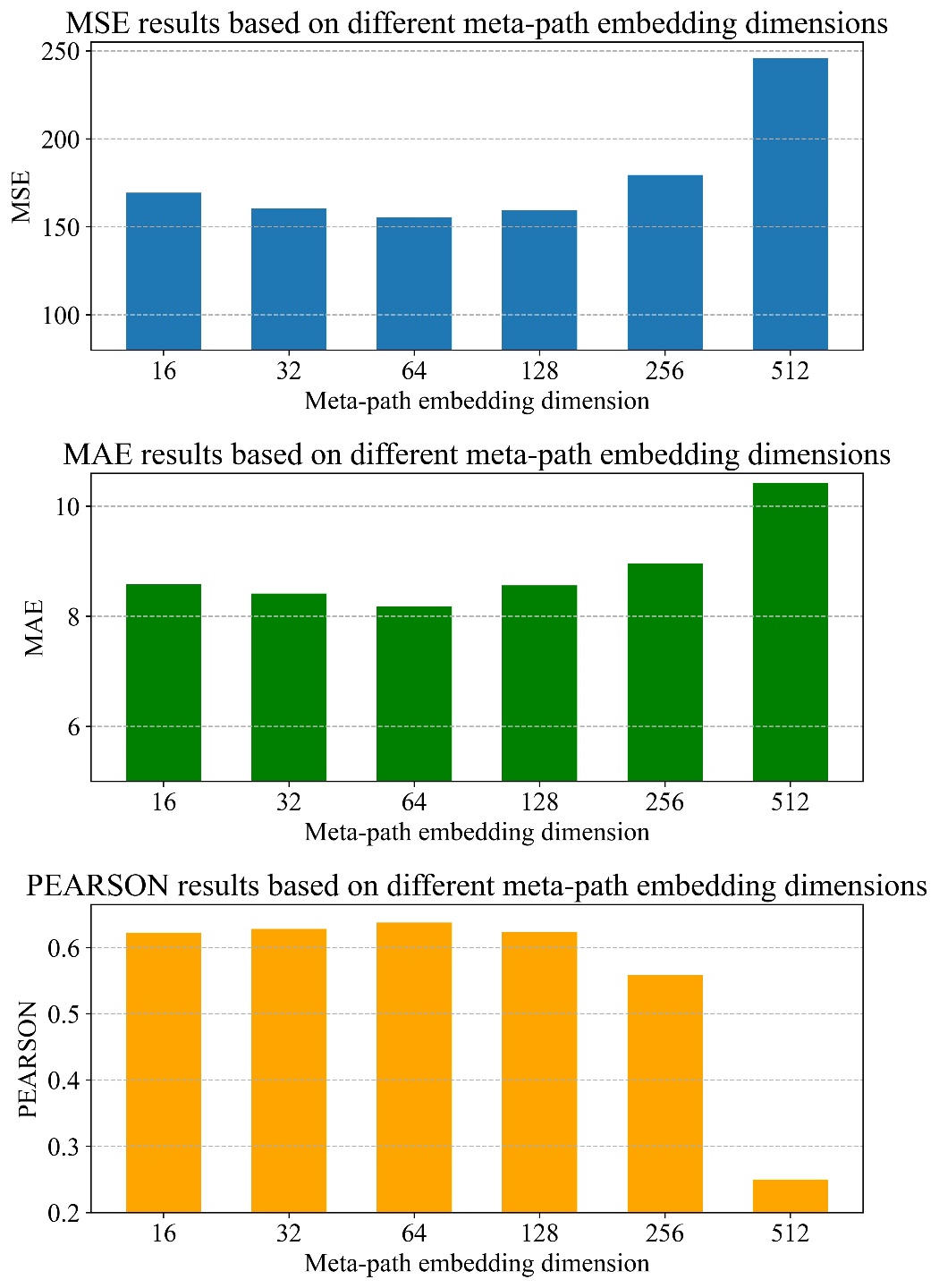


**Fig. S2.** Different model evaluation results given various meta-path embedding dimensions.

From the results, we observe that, MSE, MAE, and PEARSON gradually become better with the increase of meta-path embedding dimension, and then the performance gets worse after reaching a threshold, and 64 selected in our comparison experiments achieves the best results among these dimension choices.

**References**

Liu, Q. et al. (2021) TranSynergy: Mechanism-driven interpretable deep neural network for the synergistic prediction and pathway deconvolution of drug combinations. *PLoS computational biology*, 17(2): e1008653.

Huang, K. et al. (2020) Caster: Predicting drug interactions with chemical substructure representation. *Proceedings of the AAAI Conference on Artificial Intelligence*, 34(01): 702-709.

Peng, J. et al. (2021) An end-to-end heterogeneous graph representation learning-based framework for drug–target interaction prediction. *Briefings in Bioinformatics*, 22(5): bbaa430.

Xu, K. et al. (2018) How powerful are graph neural networks? *arXiv:1810.00826*.

1. ^a^ Centre for Computational Biology, School of Computer Science, The University of Birmingham, Edgbaston, Birmingham, B15 2TT, UK

   ^b^ State Key Laboratory of Agricultural Microbiology, Huazhong Agricultural University, Wuhan, 430070, China

   ^c^ YaoPharma Co., Ltd., 100 Xingguang Avenue, Renhe Town, Yubei District, Chongqing, 401121, China

   ^*^ To whom correspondence should be addressed. E-mail: s.he@cs.bham.ac.uk (S. He) [↑](#footnote-ref-1)
